# Arsenic Exposure and Calpain-10 Polymorphisms Impair the Function of Pancreatic Beta-Cells in Humans: A Pilot Study of Risk Factors for T2DM

**DOI:** 10.1371/journal.pone.0051642

**Published:** 2013-01-22

**Authors:** Andrea Díaz-Villaseñor, Laura Cruz, Arturo Cebrián, Raúl U. Hernández-Ramírez, Marcia Hiriart, Gonzálo García-Vargas, Susana Bassol, Monserrat Sordo, A. Jay Gandolfi, Walter T. Klimecki, Lizbeth López-Carillo, Mariano E. Cebrián, Patricia Ostrosky-Wegman

**Affiliations:** 1 Departmento de Medicina Genómica y Toxicología Ambiental, Instituto de Investigaciones Biomédicas, Universidad Nacional Autónoma de México, Mexico City, Mexico; 2 Facultad de Medicina, Universidad Autónoma de Coahuila, Torreón, Coahuila, Mexico; 3 Facultad de Medicina, Universidad Juárez del Estado de Durango, Gómez Palacio, Durango, Mexico; 4 Centro de Investigación en Salud Poblacional, Instituto Nacional de Salud Pública, Cuernavaca, Morelos, Mexico; 5 División de Neurociencias, Departamento de Neurodesarrollo y Fisiología, Instituto de Fisiología Celular, Universidad Nacional Autónoma de México, Mexico City, Mexico; 6 Department of Pharmacology and Toxicology, University of Arizona, Tucson, Arizona, United States of America; 7 Sección Externa de Toxicología, Centro de Investigación y de Estudios Avanzados del IPN (CINVESTAV), Mexico City, Mexico; 8 Departamento de Fisiología de la Nutrición, Instituto Nacional de Ciencias Médicas y Nutrición Salvador Zubirán, Mexico City, Mexico; University of Louisville, United States of America

## Abstract

The incidence of type 2 diabetes mellitus (T2DM) is increasing worldwide and diverse environmental and genetic risk factors are well recognized. Single nucleotide polymorphisms (SNPs) in the calpain-10 gene (*CAPN-10*), which encodes a protein involved in the secretion and action of insulin, and chronic exposure to inorganic arsenic (iAs) through drinking water have been independently associated with an increase in the risk for T2DM. In the present work we evaluated if *CAPN-10* SNPs and iAs exposure jointly contribute to the outcome of T2DM. Insulin secretion (beta-cell function) and insulin sensitivity were evaluated indirectly through validated indexes (HOMA2) in subjects with and without T2DM who have been exposed to a gradient of iAs in their drinking water in northern Mexico. The results were analyzed taking into account the presence of the risk factor SNPs SNP-43 and -44 in *CAPN-10*. Subjects with T2DM had significantly lower beta-cell function and insulin sensitivity. An inverse association was found between beta-cell function and iAs exposure, the association being more pronounced in subjects with T2DM. Subjects without T2DM who were carriers of the at-risk genotype SNP-43 or -44, also had significantly lower beta-cell function. The association of SNP-43 with beta-cell function was dependent on iAs exposure, age, gender and BMI, whereas the association with SNP-44 was independent of all of these factors. Chronic exposure to iAs seems to be a risk factor for T2DM in humans through the reduction of beta-cell function, with an enhanced effect seen in the presence of the at-risk genotype of SNP-43 in *CAPN-10*. Carriers of *CAPN-10* SNP-44 have also shown reduced beta-cell function.

## Introduction

Established risk factors for type 2 diabetes mellitus (T2DM), such as genetics, diet and lifestyle, do not fully explain the increase in the number of cases of this disease and environmental diabetogenic pollutants are increasingly to blame [1]. Among these environmental pollutants, inorganic arsenic (iAs) exposure is a major public health concern. An increase in the incidence and prevalence of T2DM has been observed consistently among residents exposed chronically to high iAs concentrations mainly through naturally contaminated drinking water (>100 µg/l) in countries such as Bangladesh, Taiwan and Mexico [2], [3], [4], [5], [6], [7], as well as in communities exposed to low iAs concentrations (<10 µg/l) in the United States [8]. Furthermore, in pregnant women iAs exposure has been associated with increased risk of impaired glucose tolerance at 24–28 weeks gestation and may therefore be associated with gestational diabetes [9].

Experimental evidence using a wide variety of rodent study models supports the epidemiologic data. In pancreatic beta-cells, low iAs levels (arsenite 0.5–5 µM) decrease insulin transcription and down-regulate its secretion [10] by decreasing the calcium-dependent calpain-10 proteolysis of SNAP-25 [11], a process necessary for glucose-stimulated insulin secretion [12]. Moreover, oxidative stress induced by low levels of iAs has also been shown to disturb beta-cell function, including through the induction of apoptosis [13], [14]. *In vitro* studies in adipocytes also show that iAs and its metabolites decrease insulin sensitivity [15], [16].

Rodents exposed chronically to high doses of iAs develop hyperglycemia or impaired glucose tolerance, hyperinsulinemia and/or low insulin sensitivity, as well as pancreatic stress and oxidative damage [17], [18], [19]. Additionally, a high-fat diet has been shown to act synergistically with iAs to induce glucose intolerance in mice [20]. An expert committee recently concluded that iAs exposure is associated with an increased risk for T2DM in humans; however, the evidence is still limited and there is need for additional research [21].

In the Mexican population, as well as in other populations, an increased risk of T2DM has been linked to polymorphisms of the calpain-10 gene (*CAPN-10*) [22], [23], [24]. The calcium-dependent, non-lysosomal cysteine protease calpain-10 is involved in the reorganization of the actin cytoskeleton required for both insulin exocytosis in pancreatic beta-cells [25] and insulin-stimulated GLUT4 translocation to the plasma membrane in adipocytes [26], among other functions. Interestingly, we have shown that iAs can reduce insulin secretion in beta-cells through a decrease in the proteolysis of SNAP-25 by calpain-10 [11]. Calpain-10 as part of the fusion machinery of beta-cells works as a calcium sensor regulating insulin exocytosis [12], [27], a mechanism that can be disturbed by iAs [11].

To understand the mechanism of iAs in T2DM pathogenesis more thoroughly, we performed a pilot study to evaluate insulin secretion and insulin sensitivity indirectly through validated indexes (HOMA2: a computational structural model of the feedback system of glucose and insulin in a steady state) in subjects with and without T2DM exposed to a gradient of iAs in their drinking water in northern Mexico. Because genetics and environment, as well as their interaction, play a crucial roles in T2DM onset [28], the data were analyzed taking into account risk polymorphisms in *CAPN-10* conferring risk for T2DM in Mexican-American and Mestizo-Mexican populations: SNP-43 (rs3792267), Indel-19 (rs3842570), SNP-63 (rs5030952) and SNP-44 (rs2975760) [22], [23].

## Materials and Methods

### Ethics Statement

All individuals were informed about the procedure, signed an informed consent form and answered a questionnaire. The ethics committee from the Instituto de Investigaciones Biomédicas, UNAM and the IRB from the University of Arizona approved the research protocol, and the investigation was conducted according to the Declaration of Helsinki principles.

### Study Subjects

Non-diabetic subjects and unrelated individuals with previously diagnosed T2DM were selected from the database of a study previously performed in the region [7]. Criteria for T2DM diagnosis were in accordance with those of the American Diabetes Association [29]. Subjects selected for the current study were recruited from the counties of Granada, Hidalgo, Porvenir, Purísima and San Salvador in the municipalities of Matamoros and Francisco I. Madero, Coahuila, México. The new selection criteria included age between 35 and 65 years with 30 or more years of residence in the study area, and subjects were matched by age, gender, community residence and previously reported total iAs concentration in the urine [7]. Subjects undergoing insulin treatment or diagnosed with any other illness (for example cancer and/or hepatitis) were excluded. The study was conducted with 72 subjects (40 with T2DM and 32 without T2DM). Ten of the individuals with T2DM were not receiving any hypoglycemic drug, one was receiving tolbutamide, two metformin, fifteen glibenclamide, and the remaining twelve subjects were receiving a combined hypoglycemic treatment of metformin and glibenclamide.

### Biochemical and anthropometric analysis

Age, sex, height, weight, waist circumference, and blood pressure were recorded for each donor. Blood samples were taken following an 8-hr fast. Glucose, total cholesterol, LDL-cholesterol, HDL-cholesterol, and triglyceride levels were measured in serum with enzymatic methods using commercially available colorimetric kits (Randox Laboratories Ltd., County Antrim, UK). Serum insulin concentration was determined through ELISA (Monobind, Inc., Lake Forest, CA, USA). Body mass index (BMI) was calculated using the formula weight/height^2^ (kg/m^2^).

### Analysis of iAs exposure and metabolism

Urine and drinking water samples were supplied by each donor, collected in polypropylene bottles and stored at −80°C until they were analyzed as previously reported [30], [31]. Briefly, arsenic species in urine [As^V^, As^III^, monomethylarsonic acid (MMA^V^), dimethylarsinic acid (DMA^V^) and arsenobetaine] were separated by HPLC. Arsenic concentrations in water samples and urine were analyzed by inductively coupled plasma mass spectrometry (ICPMS) utilizing for quality controls the Standard Reference Water, SMR 1640 (NIST, Gaithersburg, MD, USA) and the freeze-dried Urine Reference Material for trace elements (Clinchek-control; RECIPE Chemicals+INSTRUMENTS GmbH, Munich, Germany) for urine [30], [31].

Water consumption habits were ascertained through a standardized questionnaire and iAs consumption per day was determined by multiplying daily water consumption by the concentration of iAs in drinking water for each donor.

Arsenic metabolism efficiency was calculated using the following formulas [32]: first methylation = MMA^V^/(As^V^+As^III^), second methylation = DMA^V^/MMA^V^ and total methylation = (MMA^V^+DMA^V^)/(As^V^+As^III^).

### Beta-cell function and insulin sensitivity (%)

Beta-cell function and insulin sensitivity values (%) were determined from fasting serum glucose and insulin concentration values using the software HOMA2 (homeostatic model assessment) Calculator V2.2 developed at the University of Oxford [33]. The HOMA2 model is a computational structural model of the feedback system of glucose and insulin in the steady state (overnight fasting). The model consists of a number of nonlinear empirical equations that describe the functions of the organs and tissues involved in glucose regulation in such a way that beta-cell function (insulin secretion) and insulin sensitivity (glucose uptake) percentages can be calculated. The accuracy and precision of HOMA2 correlates highly with the “gold standard” technique of the hyperinsulinemic-euglycemic clamp and with the glucose tolerance test curve, even in subjects taking insulin secretagogues [34], [35], [36]. In six samples with out-of-range insulin values utilized by the HOMA Calculator V2.2 (2.9 to 57.6 mmol/l), it was not possible to calculate beta-cell function or insulin sensitivity percentage.

### *CAPN-10* genotyping

Genomic DNA was extracted from 1 ml of whole blood using the QIAamp DNA MINI kit (Qiagen, Valencia, CA, USA). The single nucleotide polymorphisms (SNPs) 43 (g.4852, G>A; rs3792267) and 44 (g.4841, T>C; rs2975760) were determined by site-specific Taqman assay by Design (Applied Biosystems, Foster City, CA, USA) using the following primers and probes: For SNP-43 the primers 5′-GCGCTCACGCTTGCT-3′ and 5′-CCTCACCAAGTCAAGGCTTAGC -3′ and the probes 5′-CCTTCAAAcGCCTTAC -3′ labeled with the reporter dye FAM and 5′-CACCTTCAAAtGCCTTAC-3′ labeled with the reporter dye VIC, were used. SNP-44 was amplified with the primers 5′-GCAGGGCGCTCACG-3′ and 5′-CCTCACCAAGTCAAGGCTTAGC-3′ and with the probes 5′-CCTTACTTCaCAGCAAG-3′ labeled with FAM and 5′-CCTTACTTCgCAGCAAG-3′ labeled with VIC. The cycling conditions were 95°C for 15 min followed by 40 cycles of 92°C for 15 sec and 1 min at 60°C. Indel-19 (g.7920 in/del32pb; rs3842570) was analyzed by PCR with the primers 5′-GTTTGGTTCTCTTCAGCGTGGAG-3′ and 5′-CATGAACCCTGGCAGGGTCTAAG-3′ followed by agarose gel electrophoresis to determine the size of the generated products (87 and/or 155 bp) as described [37]. Finally, SNP-63 (g.16378, C>T; rs5030952) was analyzed by direct sequencing with the primers 5′-TCGGGACACTGCTGTTAGGT-3′ and 5′CTGGCTGGAGTTTGGAGAAG-3′. The cycling conditions were 94°C for 2 min, 40 cycles of 94°C for 30 sec, 59°C for 30 sec and 72°C for 80 sec, followed by a final extension of 72°C for 5 min. The direct PCR sequencing reactions were performed using Big Dye V3.0 (Applied Biosystems, Foster City, CA, USA) as previously described [30].

Haplotype for SNPs -43, -19 and -63 was reconstructed using the PHASE program, version 2.1.1 [38], [39] and named according to the nomenclature proposed by Horikawa [22].

### Statistical Analyses

The normality of all variables was verified by skewness and kurtosis probes (STATA 9.2), followed by the Kolmogorov-Smirnov test (GraphPad Prism 5.0). All variables that did not fall within the normal distribution were Log-transformed for analysis and are indicated in each case. The statistical significance between both study groups was determined for each variable using an unpaired t-test. Variability by sex was determined using the χ^2^ test (GraphPad Prism 5.0). Deviation from Hardy-Weinberg equilibrium with Pearson's goodness-of-fit-χ^2^ test was evaluated using Finetti Generator 3.0.5 [40].

Linear regressions of Log-transformed data were performed to associate arsenic concentration excreted in urine with beta-cell function and with insulin sensitivity values (%) (GraphPad Prism 5.0).

For each group of subjects, regression analyses of Log-transformed data were carried out to determine the association between beta-cell function and SNPs in *CAPN-10* as dependent and independent variables, respectively. Associations were adjusted by iAs exposure and known risk factors for T2DM such as age, sex and BMI in a multivariate analysis. Genotypes were coded by dummy variables, three for SNP-43 corresponding to G/G, G/A and A/A alleles and two for SNP-44 analogous to T/T and T/C alleles (STATA 9.2).

In all cases, *p* values<0.05 were considered statistically significant.

## Results

### Study subjects and iAs exposure

The only clinical and biochemical characteristic significantly different between the two subject groups (type 2 diabetic and non-diabetic) was higher serum glucose in the diabetic subjects (Table 1). Both groups of subjects had been exposed to similar iAs concentrations through drinking water. The arsenic exposure gradient in the drinking water of subjects ranged from 4.0 to 145.3 µg/l (or ppb) for the non-diabetic group and 2.8 to 131.5 µg/l in the diabetic group.

**Table 1:** Clinical and biochemical characteristics and exposure to iAs in non-diabetic and diabetic subjects.

Variables		*non-diabetic subjects (n = 32)*		*Type 2 diabetic subjects (n = 40)*		
		Mean ± SD	Median (25^th^ & 75^th^)	Mean ± SD	Median (25^th^ & 75^th^)	*p*
**Sex, Male**	**(N, %)**	11, 34.4		14, 35		0.9559
**Age**	**(years)**	52.56±2.56	53.0 (44.25, 61.5)	51.85±8.77	50.0 (45.0, 59.75)	0.7406
**Weight**	**(kg)**	76.87±13.47	75.80 (68.90, 88.25)	75.33±12.65	74.80 (66.0, 85.5)	0.6202
**Height ^£^**	**(m)**	1.59±0.08	1.58 (1.54, 1.65)	1.59±0.10	1.55 (1.52, 1.67)	0.8832
**BMI**	**(kg/m^2^)**	30.58±5.89	29.65 (26.05, 34.18)	29.91±4.26	29.0 (27.3, 32.5)	0.5810
**Waist circumference**	**(cm)**	102.6±12.26	105.0 (93.5, 112.0)	103.6±10.43	103.0 (96.75, 112.0)	0.7102
**Systolic pressure ^£^**	**(mmHg)**	128.5±16.67	130.0 (113.8, 140.0)	128.3±16.33)	130.0 (120.0, 140.0)	0.9662
**Diastolic pressure ^£^**	**(mm Hg)**	81±10.46	80 (70.0, 90.0)	79.87±8.89	80.0 (70.0, 81.25)	0.6913
**Glucose ^£^**	**(mmol/l)**	5.25±0.66	5.22 (4.77, 5.79)	10.66±4.11	10.57 (6.93, 13.95)	<0.0001**
**Total cholesterol ^£^**	**(mmol/l)**	5.18±1.47	5.0 (3.99, 6.0)	5.14±1.05	5.14 (4.33, 5.63)	0.8933
**LDL-cholesterol ^£^**	**(mmol/l)**	1.66±1.12	1.52 (0.85, 2.22)	1.60±0.69	1.61 (1.22, 1.80)	0.6416
**HDL-cholesterol ^£^**	**(mmol/l)**	1.45±0.64	1.30 (1.04, 1.59)	1.25±0.39	1.20 (0.96, 1.51)	0.1565
**Triglycerides ^£^**	**(mmol/l)**	23.84±26.83	15.65 (10.79, 24.55)	22.57±16.49	18.87 (13.54, 22.13)	0.5604
**Insulin ^£^**	**(mU/l)**	14.87±10.81	11.80 (6.55, 29.95)	15.31±8.0	14.45 (8.9, 19.08)	0.3900
**Time of diagnosis of T2DM**	**(years)**			6.77±4.44		
**Arsenic in drinking water ^£^**	**(µg/l)**	78.38±29.35	72.21 (60.85, 95.11)	66.68 36.12	71.89 (48.78, 93.39)	0.0841
**Arsenic consumption ^£^**	**(µg/day)**	108.60±65.24	90.57 (70.16, 148.8)	125.90 99.04	132.70 (49.55, 160.1)	0.5470
**Total arsenic in urine**	**(µg/l)**	133.40±67.0	135.20 (81.04, 168.5)	100.90 65.21	86.92 (52.99, 133.1)	0.0443*

Data, except for sex that is expressed by the number and % of male, are represented by mean ± SD and median with 25^th^ and 75^th^ percentiles. Symbol denotes Log-transformation for analysis (**£**). BMI: body mass index. Symbols denotes statistical significance, p<0.05 (*) and p<0.0001 (**).

Despite the similarity in the concentration of iAs ingested between the two groups, total arsenic excreted in urine, a biomarker of arsenic exposure, was significantly higher in the non-diabetic subjects than in diabetic individuals (Table 1). Analysis of iAs metabolism through species in urine revealed that non-diabetic subjects have a more efficient metabolism of the metalloid, particularly in the first methylation (MMA^V^/iAs) (Table S1). This finding may explain why iAs excreted in urine was significantly higher in non-diabetic subjects than in diabetic individuals.

### Genetic characteristics of the subjects

The allele frequency distribution of at-risk polymorphisms for T2DM in *CAPN-10* (SNPs -44, -43, -63 and Indel-19) in non-diabetic and diabetic subjects is shown in Table S2. All of the SNPs were in Hardy-Weinberg equilibrium and none of the donors were homozygous for the C allele of SNP-44. Based on the reconstitution of the haplotype, only two samples from the diabetic group had the at-risk haplotype for T2DM (112/121) for SNPs -43, -19 and -63 (Table S3) previously described for the Mexican-American population [22]. Due to the low frequency of the at-risk haplotype 112/121 found in the study sample, forward genotype stratification was analyzed only with SNPs -43 and -44 because these are the SNPs most associated with T2DM phenotype [23], [41], [42], [43], [44]. The allele frequency for SNP-43 and -44 was in accordance to that previously reported for Mexicans [23].

### Association of beta-cell function with iAs exposure

**Figure 1:**
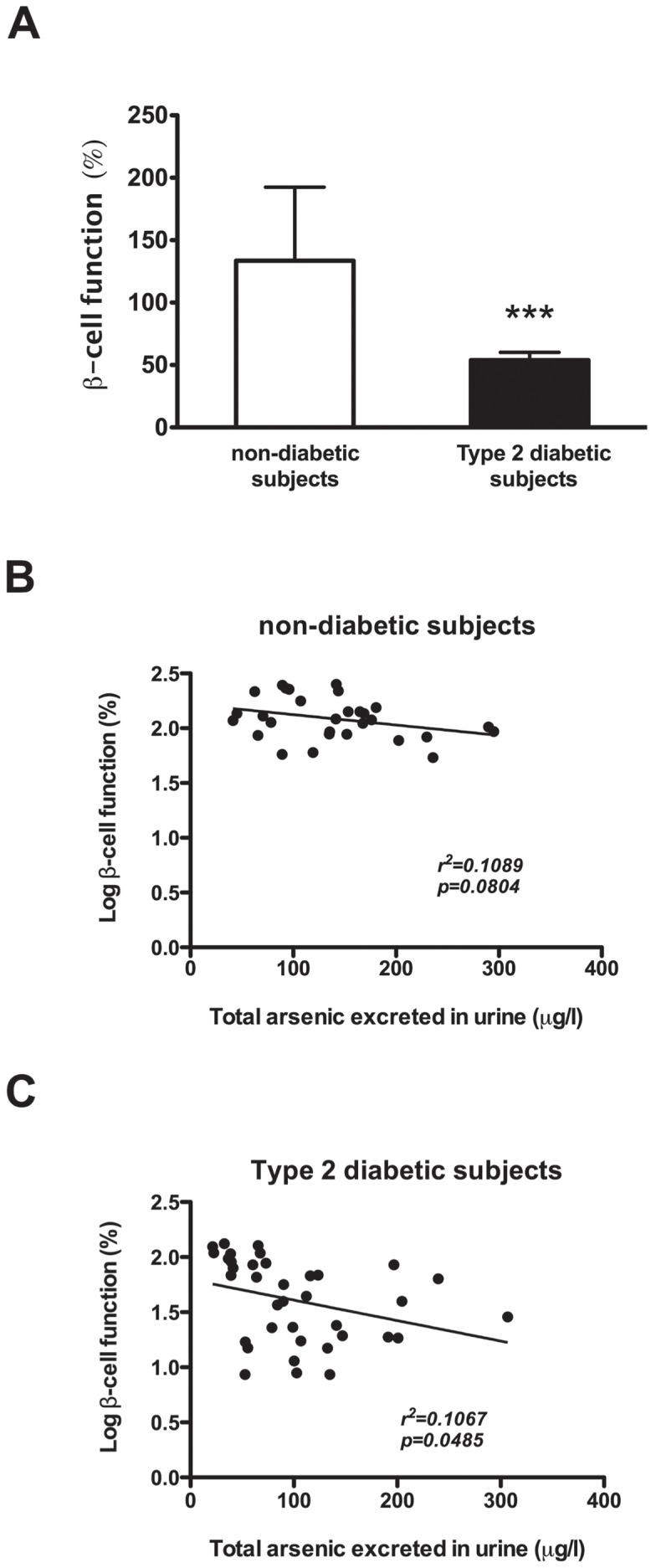
Beta-cell function (%) and association with the concentration of total arsenic excreted in urine. **A**) Beta-cell function (%) in subjects with and without T2DM; values represent mean ± SD. Symbol (***) denotes statistical significance *p*<0.0001 (data Log-transformed for analysis). **B**) Linear regression of the concentration of total arsenic excreted in urine and the % of Log-beta-cell function in non-diabetic donors, and **C**) Linear regression of the concentration of total arsenic excreted in urine and the % of Log-beta-cell function in diabetic donors.

The percentage of functional beta-cells was statistically lower in subjects with T2DM, as expected (Figure 1A). The relationship between the concentration of arsenic in urine and the function of beta-cells in the non-diabetic individuals shows an inversely proportional trend with a slope value of −0.24 but was not statistically significant (r^2^ = 0.08, *p* = 0.1441) (Figure 1B). In subjects with T2DM, an inverse association between the concentration of arsenic in urine and the level of beta-cell function was clearly demonstrated, with a slope value of −0.65 (r^2^ = 0.1067, *p* = 0.0485) (Figure 1C).

### Stratification of beta-cell function by *CAPN-10* genotype with and without consideration of iAs exposure

Because calpains, including calpain-10, are involved in the secretion of insulin and can be altered by arsenic treatment *in vitro* and because certain SNPs in this gene have been associated with T2DM, the values of the function of beta-cells were stratified by allele for SNP-43 and SNP-44.

**Figure 2:**
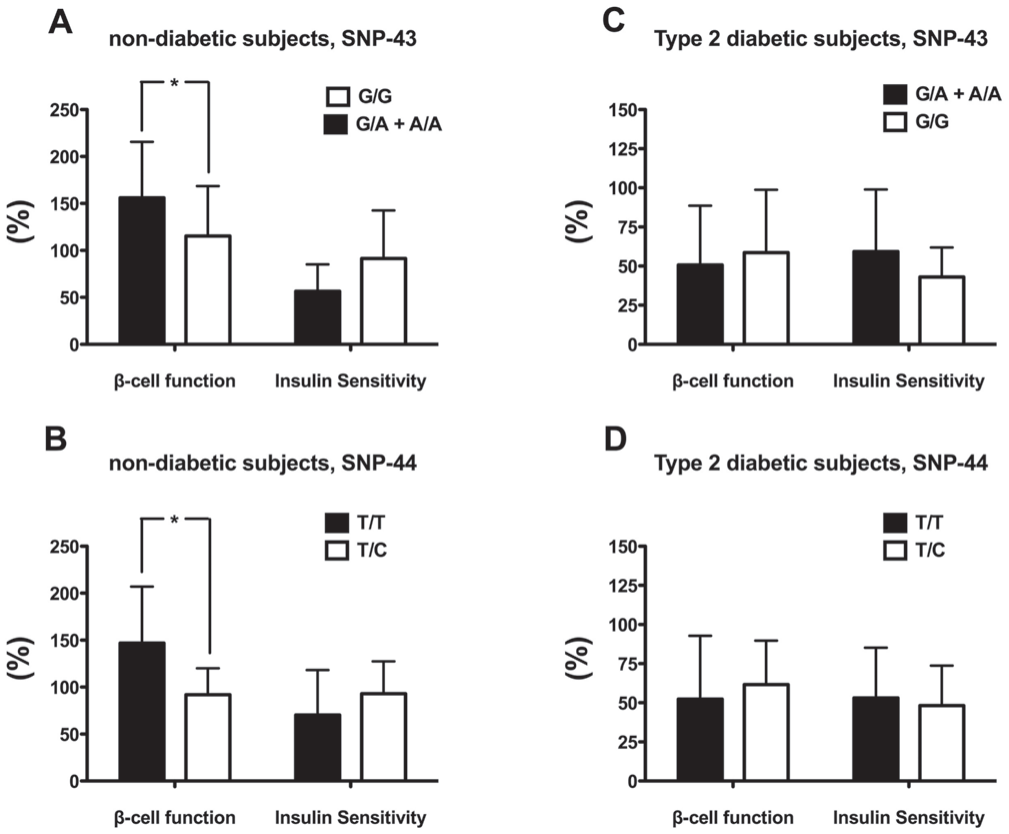
Beta-cell function and insulin sensitivity (%) stratified by *CAPN-10* genotype (SNP-43 and -44) in **A**) non-diabetic subjects, SNP-43, **B**) non-diabetic subjects, SNP-44, **C**) Type 2 diabetic subjects, SNP-43 and **D**) Type 2 diabetic subjects, SNP-44. Values represent mean ± SD. Symbol (*) denotes statistical significance *p*<0.05 between alleles (G/G *vs.* G/A+A/A for SNP-43 and T/T *vs.* T/C for SNP-44). Beta-cell function values were Log-transformed for statistical analyses.

When beta-cell function was analyzed based on the SNP-43 genotype, the subjects without T2DM with the at-risk genotype G/G had a percent of beta-cell function statistically lower than those with the genotypes G/A or A/A (Figure 2A). Similarly, when data were analyzed based on the SNP-44 genotype, the subjects without T2DM with the at-risk T/C genotype had statistically lower beta-cell function than those with the T/T genotype (individuals with the C/C genotype were not found in this study) (Figure 2B). In the group of individuals with T2DM, differences were not found between any of those with these two SNPs, as expected (Figure 2C and 2D).

The association of *CAPN-10* genotypes with beta-cell function, taking into account the exposure to iAs and established risk factors for T2DM such as age, sex, and BMI is shown in Table 2. Non-diabetic individuals with the not at-risk genotype A/A for SNP-43 had higher beta-cell function than those with the genotype G/G (*p* = 0.037); nevertheless, when the association was adjusted by arsenic excreted in urine or other known risk factors for T2DM, the association lost its significance.

**Table 2:** Univariate and multivariate association between alleles for SNPs-43 and -44 in *CAPN-10* and Log-beta cell function (%) in non-diabetic and diabetic subjects.

				Adjusted by			
				arsenic in urine		arsenic in urine, age, sex and BMI	
		*ß coefficient*	*p*	*ß coefficient*	*p*	*ß coefficient*	*p*
***Non-diabetic subjects***	**SNP-43**						
	***G/A vs A/A***	−0.34	0.226	−0.28	0.302	−0.09	0.748
	***G/G vs A/A***	−0.57	0.037*	−0.49	0.073	−0.32	0.278
	*P* for trend		0.024*		0.050		0.128
	**SNP-44**						
	***C/T vs T/T***	−0.43	0.022*	−0.44	0.014*	−0.38	0.028*
***Type 2 diabetic subjects***	**SNP-43**						
	***G/A vs A/A***	−0.02	0.964	−0.05	0.912	0.14	0.763
	***G/G vs A/A***	0.05	0.914	−0.01	0.989	0.12	0.814
	*P* for trend		0.871		0.978		0.874
	**SNP-44**						
	***C/T vs T/T***	0.47	0.211	0.56	0.156	0.57	0.160

*ß* coefficients refer to the estimated change in Log-transformed beta-cell function (%) values between each allele. Symbol denotes statistical significance p<0.05 (*).

The association of beta-cell function with SNP-44 in non-diabetic individuals also showed that subjects with the not at-risk genotype T/T for SNP-44 had higher beta-cell function in comparison to individuals with the T/C genotype (*p* = 0.024) independent of arsenic exposure (*p* = 0.014) or age, gender and BMI (*p* = 0.028). As expected, in subjects with T2DM, neither of the two SNPs showed a statistically significant association with beta-cell function (Table 2).

### Association of insulin sensitivity with arsenic exposure and *CAPN-10* genotype

**Figure 3:**
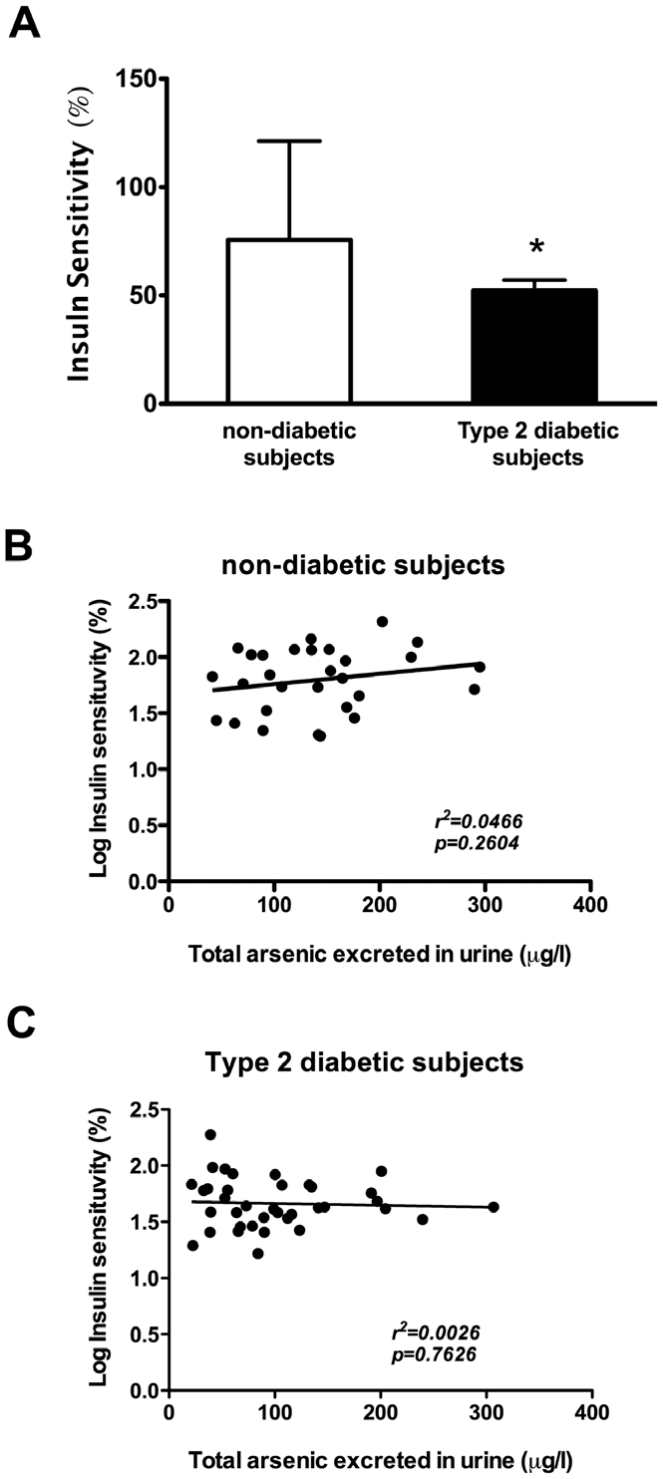
Insulin sensitivity (%) and its association with the concentration of total arsenic excreted in urine. **A**) Insulin sensitivity (%) of subjects with and without T2DM; values represent mean ± SD. Symbol (*) denotes statistical significance *p*<0.05 (data Log-transformed for analysis). **B**) Linear regression of Log-insulin sensitivity and arsenic in urine in non-diabetic subjects **C**) Linear regression of Log-insulin sensitivity and arsenic in urine in type 2 diabetic subjects.

The level of insulin sensitivity (reciprocal to insulin resistance) was statistically higher in subjects without T2DM in comparison to those with T2DM (Figure 3A). However, unlike beta-cell function, insulin sensitivity, and therefore insulin resistance, was not associated with the excretion of total arsenic in urine in either of the two analyzed groups (Figure 3B and 3C). Furthermore, insulin sensitivity was also stratified by alleles for SNP-43 and SNP-44 because calpain-10 also participates in insulin-dependent glucose uptake [26], [45], [46]. Insulin sensitivity measured through HOMA2 was not significantly influenced by these SNPs (Figure 2).

## Discussion

Epidemiological studies in humans [2], [3], [4], [5], [6], [7], [8], [9] as well as *in vitro* and *in vivo* studies in rodents [10], [11], [13], [14], [16], [17], [18], [19], [20], have been conducted to determine if iAs exposure is a risk factor for T2DM. Analyses of human samples from subjects exposed to iAs are lacking, especially studies of the effects of the metalloid in tissues such as pancreas and liver. Therefore, pilot studies such as this one are very valuable, even though the number of subjects involved was small. Although evidence shows that high iAs exposure and T2DM are significantly associated, additional research is needed to determine whether the relationship is causal as proposed by The National Institute of Environmental Health Sciences (NIEHS), Division of the National Toxicology Program (NTP) this past year [21]. Moreover, evidence showing that iAs exposure is associated with an increased risk for T2DM in humans is still limited [21], and until now, studies that try to explain the mechanism of how arsenic acts as a risk factor for T2DM have been carried out in rodents or in *in vitro* studies but never in humans; therefore, this pilot study is relevant. The data obtained from this work support the previous findings from studies carried out in non-human subjects, especially those focusing on beta-cell dysfunction.

In this pilot study, we measured the interaction of iAs on glucose metabolism in humans using non-invasive methods. We also analyzed the association between this environmental metalloid and genetic factors for the disease. Our results show that iAs indeed does affect the pathogenesis of T2DM, particularly in disturbing beta-cell function, and this in turn is also associated with SNPs found in *CAPN-10*, a susceptibility gene for T2DM in certain populations, including Mexicans [22], [23], [24].

It was expected that daily arsenic consumption and levels of arsenic excreted in the urine would be correlated. Nevertheless, non-diabetic subjects consumed less iAs but had greater concentrations of arsenic in the urine than subjects with T2DM. This phenomenon could be due to differences in arsenic metabolism or, to a less extent, to an inaccuracy in self-reporting of water consumption habits.

The relationship between the concentration of arsenic in urine and the function of beta-cells in diabetic and non-diabetic subjects show the same trend, but the association was statistically significant only in the diabetic subjects. These data suggest that iAs exposure is a risk factor for T2DM rather than a direct inducer of the disease. Failure of beta-cell function is necessary for progression from insulin resistance to T2DM; nevertheless, it is not the only event that is required because many other risk factors are also needed for the development of the diabetic state [47]. In this work, we noticed that, in subjects without T2DM, iAs exposure was associated with a decrease in the function of beta-cells, but this was not sufficient for the development of T2DM. Other risk factors would need to exert their actions, indicating a multifactorial metabolic disruption in the organism. Another possibility may be that the beta-cells of diabetic subjects are extremely sensitive to iAs exposure; however, in this study it was not possible to determine this as a cause (i.e., beta-cells are impaired directly by iAs) or a consequence (i.e., the function of beta-cells from diabetic subjects is deficient and thus cells are more susceptible to the effects of iAs). However, *in vitro* studies have shown that iAs impairs insulin secretion, leading to beta-cell insensitive to glucose [10], [11]. It could also be possible that by increasing the sample size, a clearer association could be determined between iAs exposure and beta-cell function in non-diabetic subjects.

T2DM is a complex disease, and an interaction between environmental factors and genetic components occurs in the pathogenesis of the disease. In this work we evaluated the influence of SNPs in *CAPN-10* on beta-cell dysfunction. Other genes have been associated with T2DM such as *TCF7L2*, *SLC30A8*, *HHEX*, *CDKAL1*, *IGF2BP2*, *CDKN2A/B*, *PPARG*, *KCNJ11*, *KCNQ1 and MTNR1B*, as determined through genome-wide association studies (GWAS) [48], [49]. However, this study analyzed SNPs in *CAPN-10* because these have been associated with T2DM in the Mexican population [23] and calpain-10 participates in both glucose-stimulated insulin secretion in beta-cells [25] and in insulin-stimulated GLUT4 translocation to the plasma membrane in adipocytes [26]. Moreover, we previously showed that iAs can reduce insulin secretion in beta-cells through a decrease in the proteolysis of SNAP-25 by calpain-10 [11].

SNP-43 in *CAPN-10* is involved in many of the phenotypes associated with T2DM, such as insulin resistance, lipogenesis, insulin secretion and microvascular function [41], [42], [43], [44], while SNP-44 has been associated with increase risk for T2DM in Mexican population [23] and with other populations as evaluated through meta-analysis [24]. SNP-44 has also been associated with alterations in the pattern of the oral glucose tolerance curve [50] and is in linkage disequilibrium with the missense variant T504A (SNP-110), which unlike the other SNPs, is located in the open reading frame of CAPN-10 [22], [23].

Beta-cell function was lower in subjects without T2DM that carried the at-risk genotypes G/G at SNP-43 and T/C at SNP-44. In subjects with T2DM, the influence of the genotype on beta-cell function was not evident because the level of function of these pancreatic cells is already altered due to the disease *per se* and to all the factors that contributed to the onset of it; therefore, the influence of the genotype has been lost. Once the disease has been established, which not only involves the dysfunction of beta-cells but also other events [47], it is likely that at-risk variants in *CAPN-10* are not more associated with the level of beta-cell function, even when iAs exposure occurs. Instead, other mechanisms, such epigenetic modifications, may be taking place, as has been revealed in a genome wide site-specific DNA methylation study conducted in peripheral blood lymphocyte DNA of 16 individuals, half with established elevated levels of iAs exposure and showing signs of arsenicosis (skin lesions). The investigators in this study found that epigenetic changes in genes were associated with arsenic-induced diseases, including T2DM [51].

From the analysis of beta-cell function stratified by *CAPN-10* genotypes through multivariate analysis, it can be postulated that the at-risk heterozygote allele T/C in SNP-44 is associated with decreased beta-cell function independently of arsenic exposure or other risk variables for T2DM, such as age, sex and BMI. On the contrary, the risk for the development of T2DM with the G/G genotype at SNP-43 depends upon intrinsic and environmental factors, including iAs exposure.

As mentioned above, for T2DM to occur, other components, such as insulin resistance, are also needed. In contrast to *in vitro* findings in the literature in which arsenic induces insulin resistance in adipocytes [15], [16], in this pilot study insulin sensitivity was not associated with iAs exposure or with at-risk alleles in *CAPN-10*. This lack of correlation may be due to the relationship between glucose and insulin in the basal state (fasting) in the HOMA analysis, which reflects the balance between hepatic glucose output and insulin secretion that is maintained by a feedback loop between the liver and the pancreas [35], and less with muscle or adipose tissue insulin sensitivity, which are the organs where glucose uptake occurs in an insulin-dependent manner. In fact, the studies of *in vitro* arsenic effects were conducted mostly in adipocytes and not in hepatic cells. Additionally, it cannot be ruled out that the lack of correlation was influenced by metformin treatment in diabetic subjects (50%) or by the small sample size.

In a recent cross-sectional study conducted in Zimapán, México, in addition to the prevalence of diabetes being positively associated with iAs in drinking water and with the concentration of its metabolite dimethylarsinous acid in urine, an inverse association (*p*<0.01) between HOMA-insulin resistance and iAs exposure was also shown, contrary to our results and to *in vitro* findings [52]. Nonetheless, more studies in humans are needed to understand more deeply this phenomenon.

Taken together, these results provide evidence in humans that support the idea that iAs is a risk factor for T2DM through the disturbance of beta-cell function. As is known, individuals with beta-cells unable to sustain increased insulin secretion to compensate for insulin resistance will develop T2DM, and thus inadequate beta-cell function is essential to the course of the disease [28]. Here we show that both iAs exposure, as well as presence of the at-risk alleles of SNP-43 and SNP-44 in *CAPN-10*, a protein that plays an active role in insulin secretion as part of the machinery of the secretion complex [12], have an adverse effect on beta-cell function. It is possible that beta-cell failure may occur earlier and/or in an exacerbated manner in individuals with the at-risk genotypes in SNP-44 (T/C) and/or in subjects with the at-risk genotypes in SNP-43 (G/G) and exposure to iAs.

Epidemiological associations between chemical exposure and disorders of glucose metabolism are increasing every day. According to The Environmental Protection Agency (EPA), an endocrine disrupting chemical (EDC) is defined as an exogenous agent that interferes with the production, release, transport, metabolism, binding, action, or elimination of natural hormones in the body responsible for the maintenance of homeostasis, reproduction, development and/or behavior [1]. iAs as well as other pollutants, such as polychlorinated biphenyls, organochlorine pesticides, dioxins, and bisphenol A, among others [1], [53], [54], need to be seriously taken into account as environmental factors that increase the risk for T2DM.

Finally, we believe this study improves the current understanding of T2DM, particularly by adding evidence of iAs acting as a diabetogen in humans and contributing to the fact that environmental factors other than diet and exercise, together with genetic influences, are involved in the development of the disease. In additional to epidemiological studies, it is very important to conduct biological analyses in humans and to find the way to eliminate efficiently iAs exposure.

## Supporting Information

Table S1 **Arsenic metabolism.** Data are represented by mean ± SD and median with 25^th^ and 75^th^ percentiles. Symbol denotes Log-transformation for analysis (**^£^**). Symbol (*) denotes statistical significance p<0.05.
(DOC)

Table S2 **Allelic frequencies of** ***CAPN-10*** **polymorphisms (SNP-44, -43, -63 and Indel-19) in non-diabetic and diabetic subjects.** *p* values of the deviation from Hardy-Weinberg equilibrium were 0.419, 0.879, 0.339 and 0.945 for SNP-44, SNP-43, Indel-19 and SNP-63, respectively for non-diabetic subjects. For diabetic subjects the *p* values were 0.544, 0.610, 0.436 and 0.376 for SNP-44, SNP-43, Indel-19 and SNP-63, respectively.
(DOC)

Table S3 **SNP-43, Indel-19 and SNP-63 haplotype frequency in non-diabetic and diabetic subjects.** The nomenclature for haplotype is as follows: SNP-43, allele 1, G, allele 2, A; Indel-19, allele 1, 2 repeats of 32 bp sequence, allele 2, 3 repeats; SNP-63, allele 1, C, allele 2, T. The haplotype associated with T2DM is 112/121 (¥), denoting G/G for SNP43, 2 repeats/3 repeats for Indel-19 and T/C for SNP-63.
(DOC)
