# Supplementary material for: Arsenic Exposure and Calpain-10 Polymorphisms Impair the Function of Pancreatic Beta-Cells in Humans: A Pilot Study of Risk Factors for T2DM
Source: PLoS One. 2013 Jan 22;8(1):e51642. doi: 10.1371/journal.pone.0051642 (PMC3551951; doi:10.1371/journal.pone.0051642)
Supplement: Table S1 — Arsenic metabolism. Data are represented by mean ± SD and median with 25th and 75th percentiles. Symbol denotes Log-transformation for analysis (£). Symbol (*) denotes statistical significance p<0.05. (DOC) [file pone.0051642.s001.doc]

**Table S1.**

| **Methylation of As metabolites** | | ***non-diabetic subjects (n=32)*** | | | | ***Type 2 diabetic subjects (n=40)*** | | |  | |
| --- | --- | --- | --- | --- | --- | --- | --- | --- | --- | --- |
|  |  | | **Mean** ± **SD** | | **Median (25^th^ & 75^th^)** | **Mean** ± **SD** | | **Median (25^th^ & 75^th^)** | | ***p*** |
| **First methylation:** | **MMA^V^/Asi ^£^** | 1.07 ±0.34 | | 1.02 (0.88, 1.4) | | 0.92 ±0.38 | 0.82 (0.66, 1.1) | | **0.0495*** | |
| **Second methylation:** | **(DMA^V^/MMA^V^) ^£^** | 6.28 ±2.68 | | 5.97 (3.71, 8.52) | | 7.21 ±4.2 | 7.36 (4.48, 8.37) | | 0.3915 | |
| **Total methylation:** | **DMA^V^+MMA^V^/Asi ^£^** | 7.73±4.36 | | 5.95 (4.85, 9.21) | | 7.16±3.87 | 5.92 (4.65, 9.64) | | 0.3028 | |
