# Supplementary material for: Arsenic Exposure and Calpain-10 Polymorphisms Impair the Function of Pancreatic Beta-Cells in Humans: A Pilot Study of Risk Factors for T2DM
Source: PLoS One. 2013 Jan 22;8(1):e51642. doi: 10.1371/journal.pone.0051642 (PMC3551951; doi:10.1371/journal.pone.0051642)
Supplement: Table S2 — Allelic frequencies of CAPN-10 polymorphisms (SNP-44, -43, -63 and Indel-19) in non-diabetic and diabetic subjects. p values of the deviation from Hardy-Weinberg equilibrium were 0.419, 0.879, 0.339 and 0.945 for SNP-44, SNP-43, Indel-19 and SNP-63, respectively for non-diabetic subjects. For diabetic subjects the p values were 0.544, 0.610, 0.436 and 0.376 for SNP-44, SNP-43, Indel-19 and SNP-63, respectively. (DOC) [file pone.0051642.s002.doc]

**Table S2.**

|  | | ***non-diabetic subjects (n=32)*** | | ***Type 2 diabetic subjects (n=40)*** | |
| --- | --- | --- | --- | --- | --- |
| **SNPs in *CAPN-10*** | | **N** | **Frequency** | **N** | **Frequency** |
| ***SNP-44*** | |  |  |  |  |
| **Alleles** | **T** | 56 | 0.875 | 73 | 0.913 |
|  | **C** | 8 | 0.125 | 7 | 0.088 |
| **Genotype** | **T/T** | 24 | 0.750 | 33 | 0.825 |
|  | **T/C** | 8 | 0.250 | 7 | 0.175 |
|  | **C/C** | 0 | 0.000 | 0 | 0.000 |
| ***SNP-43*** | |  |  |  |  |
| **Alleles** | **G** | 45 | 0.703 | 51 | 0.638 |
|  | **A** | 19 | 0.297 | 29 | 0.363 |
| **Genotype** | **G/G** | 16 | 0.500 | 17 | 0.425 |
|  | **G/A** | 13 | 0.406 | 17 | 0.425 |
|  | **A/A** | 3 | 0.094 | 6 | 0.150 |
| ***Indel-19*** | |  |  |  |  |
| **Alleles** | **3 repeats (3R)** | 42 | 0.656 | 47 | 0.588 |
|  | **2 repeats (2R)** | 22 | 0.344 | 33 | 0.413 |
| **Genotype** | **3R/3R** | 15 | 0.469 | 15 | 0.375 |
|  | **3R/2R** | 12 | 0.375 | 17 | 0.425 |
|  | **2R/2R** | 5 | 0.156 | 8 | 0.200 |
| ***SNP-63*** | |  |  |  |  |
| **Alleles** | **C** | 53 | 0.828 | 62 | 0.775 |
|  | **T** | 11 | 0.172 | 18 | 0.225 |
| **Genotype** | **C/C** | 22 | 0.688 | 25 | 0.625 |
|  | **C/T** | 9 | 0.281 | 12 | 0.300 |
|  | **T/T** | 1 | 0.031 | 3 | 0.075 |
