# Supplementary material for: Arsenic Exposure and Calpain-10 Polymorphisms Impair the Function of Pancreatic Beta-Cells in Humans: A Pilot Study of Risk Factors for T2DM
Source: PLoS One. 2013 Jan 22;8(1):e51642. doi: 10.1371/journal.pone.0051642 (PMC3551951; doi:10.1371/journal.pone.0051642)
Supplement: Table S3 — SNP-43, Indel-19 and SNP-63 haplotype frequency in non-diabetic and diabetic subjects. The nomenclature for haplotype is as follows: SNP-43, allele 1, G, allele 2, A; Indel-19, allele 1, 2 repeats of 32 bp sequence, allele 2, 3 repeats; SNP-63, allele 1, C, allele 2, T. The haplotype associated with T2DM is 112/121 (¥), denoting G/G for SNP43, 2 repeats/3 repeats for Indel-19 and T/C for SNP-63. (DOC) [file pone.0051642.s003.doc]

**Table S3**

|  | ***Non-diabetic subjects (n=32)*** | | ***Type 2 diabetic subjects (n=40)*** | |
| --- | --- | --- | --- | --- |
| **Haplotype** | **N** | **Frequency** | **N** | **Frequency** |
| **111/111** | 2 | 0.063 | 5 | 0.125 |
| **111/112** | 3 | 0.094 | 3 | 0.075 |
| **111/221** | 3 | 0.094 | 7 | 0.175 |
| **112/121^¥^** | 0 | 0 | 2 | 0.050 |
| **112/221** | 3 | 0.094 | 2 | 0.050 |
| **112/222** | 1 | 0.031 | 0 | 0 |
| **121/111** | 5 | 0.156 | 4 | 0.100 |
| **121/121** | 5 | 0.156 | 1 | 0.025 |
| **121/122** | 1 | 0.031 | 0 | 0 |
| **121/221** | 4 | 0.125 | 5 | 0.125 |
| **121/222** | 2 | 0.063 | 2 | 0.050 |
| **122/112** | 0 | 0 | 2 | 0.050 |
| **122/222** | 0 | 0 | 1 | 0.025 |
| **221/221** | 3 | 0.094 | 3 | 0.075 |
| **221/222** | 0 | 0 | 3 | 0.075 |
